# HTLV‐1 infection altered expression of CCR2, CXCR2, eNOS genes, and oxidative stress in aorta and heart of male mice

**DOI:** 10.14814/phy2.70409

**Published:** 2025-06-10

**Authors:** Saeed Niazmand, S. A. Rahim Rezaee, Jamshid Gholizadeh Navashenaq, Nema Mohamadian Roshan, Mohsen Ghoryani, Houshang Rafatpanah, Maryam Mahmoudabady, Yousef Baghcheghi, Maryam Paseban, Mahdiyeh Hedayati‐Moghadam

**Affiliations:** ^1^ Department of Physiology, Faculty of Medicine Mashhad University of Medical Sciences Mashhad Iran; ^2^ Immunology Research Center, Inflammation and Inflammatory Diseases Division Mashhad University of Medical Sciences Mashhad Iran; ^3^ Noncommunicable Diseases Research Center Bam University of Medical Sciences Bam Iran; ^4^ Student Research Committee, School of Medicine Bam University of Medical Sciences Bam Iran; ^5^ Department of Pathology, Ghaem Hospital Mashhad University of Medical Sciences Mashhad Iran; ^6^ Department of Laboratory Sciences, School of Paramedical Sciences Torbat Heydariyeh University of Medical Sciences Torbat Heydariyeh Iran; ^7^ Research Center of Advanced Technologies in Medicine Torbat Heydariyeh University of Medical Sciences Torbat Heydariyeh Iran; ^8^ Bio Environmental Health Hazards Research Center Jiroft University of Medical Sciences Jiroft Iran; ^9^ Student Research Committee Jiroft University of Medical Sciences Jiroft Iran; ^10^ Innovative Medical Research Center and Department of Physiology, Faculty of Medicine Mashhad Medical Science Islamic Azad University Mashhad Iran; ^11^ Department of Physiology, School of Medicine Jiroft University of Medical Sciences Jiroft Iran

**Keywords:** aorta, heart, HTLV‐1, inflammation, oxidative stress

## Abstract

Viral infections are associated with the disruption of oxidative stress and the progression of inflammatory mechanisms that play pivotal roles in cardiovascular diseases. In the present study, several inflammatory and oxidative stress markers were examined in HTLV‐1‐infected male BALB/c mice. Twenty BALB/c mice were divided into two groups: the HTLV‐1‐infected group and the control group. Two months later, samples were collected from blood, aorta, heart, spleen, and lymph nodes. Finally, the levels of various plasma markers (lipid profile, creatine phosphokinase, nitric oxide, GSH, and total thiol), oxidative stress markers (SOD and CAT activity, MDA and total thiol levels), chemokine receptors genes expression (CCR2, CXCR2, CCR1) and eNOS expression in aortic and heart tissues, as well as histopathological changes in the heart, were evaluated. Plasma triglyceride, creatine phosphokinase, nitric oxide, and aorta malondialdehyde levels in the HTLV‐1‐infected group were higher than those in the control group. In contrast, total thiol levels in plasma, heart, and aorta, plasma glutathione levels, and the activities of superoxide dismutase and catalase were lower compared to the control group. The expression of CCR2 and CXCR2 was elevated in the aorta of the HTLV‐1‐infected group, while eNOS expression was reduced in both aortic and heart tissues. HTLV‐1 may contribute to inflammatory responses and oxidative stress in cardiovascular tissues.

## 1 INTRODUCTION

HTLV‐1 infection is endemic in Central Africa, regions of South America, Caribbean islands, Australo‐Melanesians, southwestern Japan, and Khorasan Razavi (Gessain & Cassar, 2012). The prevalence of HTLV‐1 infection in the total population of Khorasan Razavi is approximately 2.3%–3% (Shoeibi et al., 2013). HTLV‐1 infection is associated with various diseases, including arthropathy, adult T cell leukemia (ATL), HTLV‐1 infection‐related dermatitis (IDH), HTLV‐1 associated myelopathy/tropical spastic paraparesis (HAM/TSP), Sjögren's syndrome, Behçet's disease, thyroiditis, optic neuritis, alveolitis, and sensorimotor polyneuropathy (Farid Hosseni et al., 2013; Paigen et al., 1990).

There are documented associations between viral infection and cardiovascular diseases (Fong, 2009; Hemmat et al., 2018). Farid Hosseini et al. reported that the incidence of cardiovascular disorders is more pronounced in HTLV‐1 carriers compared to healthy individuals (Farid Hosseni et al., 2013). Additionally, Layegh reported that the thickness of carotid intima‐media (IMT), an indicator of atherosclerosis, is greater in HTLV‐1 carriers than in healthy individuals (Layegh et al., 2014). The occurrence of cardiac failure in ATL patients has been demonstrated through pathological examinations (O'Mahony et al., 2008; Farid Hosseni et al., 2013). Koizumi et al. (2005) showed that HTLV‐1 carriers affected by rheumatoid arthritis are more susceptible to atherosclerosis (Koizumi et al., 2005).

HTLV‐1 infection can disrupt the oxidative‐antioxidative balance and increase the production of reactive oxygen species (ROS), leading to a state of oxidative stress (Kinjo et al., 2010; Takahashi et al., 2013). Accordingly, Shomali et al. reported that the natural total antioxidant capacity (TAC) is diminished in HTLV‐1‐infected patients (Shomali et al., 2015). More recently, our study demonstrated that HTLV‐1 infection is associated with increased oxidative stress in various tissues of mice, including the hippocampus and cerebral cortex (Moghadam et al., 2018), lung (Hedayati‐Moghadam et al., 2021) kidney, and liver (Niazmand et al., 2022).

Several studies have indicated that nitric oxide (NO), alongside oxidative stress, plays a significant role in the pathogenesis of atherosclerosis and cardiovascular disorders (Cachofeiro et al., 2008; Förstermann et al., 2017; Hulsmans & Holvoet, 2010; Tousoulis et al., 2012). The properties of NO in regulating vascular cell proliferation, smooth muscle contraction, vascular permeability, and coagulation are essential for maintaining cardiovascular homeostasis (Tousoulis et al., 2012). Disruption in vascular NO synthesis leads to endothelial dysfunction (Farah et al., 2018), which is considered an early marker of atherosclerosis and occurs prior to clinical evidence of atherosclerotic plaque development (Gimbrone Jr & García‐Cardeña, 2016).

The presence of ROS alone is insufficient for the progression of atherosclerosis. A close connection between inflammatory factors and oxidative stress is necessary for the advancement of atherosclerosis. The activation of immune‐inflammatory factors initiates complex molecular mechanisms, resulting in vascular and immune responses such as endothelial cell activation, smooth muscle cell proliferation, and the infiltration, activation, and adhesion of white blood cells (WBC) (Arrigo, 1999; Cachofeiro et al., 2008; Hulsmans & Holvoet, 2010; Li et al., 2014). Various studies have shown that in underlying infectious disease, T‐cell activation and subsequent inflammatory factors are responsible for atherosclerosis, rather than metabolic risk factors (Bots et al., 1997; Farid Hosseni et al., 2013; Pignoli et al., 1986). Oxidative stress induces production and secretion of cytokines such as interleukin (IL)‐1β, tumor necrosis factor (TNF)‐α, monocyte chemoattractant protein (MCP)‐1, IL‐6, and IL‐8 (Li et al., 2014). Furthermore, previous research has reported an increase in the production of interferon (IFN)‐γ, IL‐2, IL‐4, as well as IL‐6, TNF‐α, IL‐1α, and IL‐1β in the cerebrospinal fluid (CSF) and blood of HTLV‐1 infected individuals (Rafatpanah et al., 2007; Santos et al., 2004). The expression of CCR1 (Wilcox et al., 1994), CCR2 (Zhang et al., 2011), and CXCR2 (Boisvert et al., 2006) which are vascular proatherogenic chemokine receptors, increases through inflammatory responses (Weber et al., 1999), cytokine secretion (Loetscher et al., 1996), and low‐density lipoprotein (LDL‐cholesterol) oxidation (Loetscher et al., 1996). Similarly, an increase of CCR2 expression on endothelial cells and macrophages of ApoE‐KO mice undergoing atherogenesis has been observed (Zhang et al., 2011).

Based on previous clinical reports indicating that HTLV‐1 infection may be linked to cardiovascular disorders, the present study is designed to demonstrate the effects of HTLV‐1 infection on oxidative stress, the gene expression of CCR1, CCR2, CXCR2, endothelial NO synthase (eNOS), inflammatory NO synthase (iNOS) in aortic and heart tissues, as well as the lipid profile, glutathione (GSH), and creatine phosphokinase (CK) in the plasma of male BALB/c mice.

## 2 MATERIAL METHODS

### 2.1 Cells

The MT‐2 cell line (ECACC 08081401), infected with Human T‐cell leukemia virus type 1, was established by co‐culturing normal human cord blood leukocytes with leukemic T‐cells from a patient with acute T‐cell leukemia (Miyoshi et al., 1981). These MT‐2 cells were cultured in RPMI 1640 medium (Caisson, USA), supplemented with 10% fetal bovine serum (FBS), 1% L‐alanyl‐L‐glutamine (Gln), and a penicillin–streptomycin mixture (0.1%), all sourced from Gibco Company, UK.

### 2.2 Animals

This study utilized 20 adolescent male BALB/c mice, aged 4–6 weeks and weighing approximately 20–30 g, procured from the Mashhad University of Medical Sciences animal facility. The BALB/c mice were housed under standard conditions, including a temperature of 23 ± 1°C, 12‐h light/dark cycle, and free access to dry food (chow) and water. All experimental procedures were conducted in accordance with the guiding principles of animal care and use, as approved by the Mashhad University of Medical Research Sciences Ethical Commission (IR.MUMS.fm.REC.1394.171). On the first day of the study, intraperitoneal (IP) injections of saline solution and MT‐2 cells were administered. Mice were then kept without any further treatment. Randomly, ten mice received saline solution, serving as the control group, while ten mice received 10^6^ MT‐2 cells, designated as an HTLV‐1‐infected group.

### 2.3 Preparation of the samples

After 2 months, 30 mg/kg ketamine (Alfasan, the Netherlands) was administered (IP) to the mice in both groups, and peripheral blood was collected from the heart apex. Peripheral blood mononuclear cells (PBMCs) were isolated using Ficoll–Hypaque (Gibco, U.K) and plasma was obtained from ethylenediaminetetraacetic acid (EDTA)‐blood. Additionally, a lysis buffer containing KHCO3 (10 mM), EDTA (25 mM), and NH4Cl (100 mM) used to eliminate existing red blood cells in the spleen. Finally, splenocytes, PBMCs, and mesenteric lymph nodes were immediately refrigerated at −20°C for subsequent HTLV‐1 proviral DNA load (PVL) analysis. The isolated plasma was used for measurements of lipids (Rifai et al., 1999) and CK using commercial kits (Pars Azmoon, Iran). The heart apex was dissected and preserved in 10% formalin for pathological studies. The remaining parts of the aorta and heart tissue (excluding the apex) were divided into two sections: one part was immediately stored at −20°C for biochemical analyses, and the other part was placed in RNAlater solution for molecular studies.

### 2.4 Evaluation of HTLV‐1 infection

Total DNA was extracted from various tissues, including PBMCs, splenocytes, and mesenteric lymph nodes, using a DNA extraction kit from Genet Bio, South Korea. The HTLV‐1 proviral DNA load (PVL) was assessed to confirm infection status. Real‐time PCR was performed with a reaction mixture that included 2 μL of extracted DNA, 1 μL of primers at a concentration of 0.2 μmol/L, 1X TaqMan probe, 2 μL of distilled water, and 5 μL of TaqMan Master Mix (2X), utilizing a Rotorgen Q 6000 machine from Corbett Research, Australia. The quantities of HTLV‐1 DNA and the reference gene albumin were measured against standard curves. The proviral load per 10^4^ cells was calculated as follows: (number of HTLV‐1 DNA copies / number of albumin DNA copies/2) × 10^4^. For quantifying HTLV‐1 proviral DNA load, specific forward (SK110, nucleotides 4758–4779) and reverse (SK111, nucleotides 4943–4920) primers, along with the HTLV‐1 TaqMan probe, were employed (Table 1) (Moghadam et al., 2018).

**TABLE 1:** List of the sequence of primers and probs of HTLV‐1 DNA, Albumin, eNOS, iNOS, CCR2, CXCR2, and B2MG genes.

Gene	Forward primer	Reverse primer	Prob
HTLV‐1‐DNA	5′‐CCCTACAATCCAACCAGCTCAG‐3′	5′‐GTGGTGAAGCTGCCATCGGGTTTT‐3′	5′‐FAM‐CTT TACTGACAAACCCGACCTACCC ATGGA‐BHQ1‐3′
Albumin	5′‐CCTTGTCACTAGATGCAAAG‐3′	5′‐GACCATACGTGAAGACCTAA‐3′	5′‐FAM‐CACATCACAACCACAACCTTCTCAG‐BHQ1‐3′
eNOS	5′‐GTTCAGCCATCACAGTGTTC‐3′	5′‐GGGGCAGAGTGAAGAGTTC‐3′	5′‐HEX‐TTGATGTGCTGCCCCTGTTA‐BHQ1‐3′
iNOS	5′‐AGGACATTAACAACAACGTGA‐3′	5′‐TGGTGAAGAGTGTCATGCAA‐3′	5′‐FAM‐AGCTGCATGTGACATCGAC‐BHQ1‐3′
CCR1	5′‐AAGGCCCAGAAACAAAGTCT‐3′	5′‐CAGTCTTTTGGCATGGAGT‐3′	5′‐HEX‐TCACAGAAGCCTACCCCACA‐BHQ1‐3′
CCR2	5′‐GAAGAGGGCATTGGATTCAC‐3′	5′‐GCTCCAATTTGCTTCACACT‐3′	5′‐FAM‐ACCTCAGTTCATCCACGGCA‐BHQ1‐3′
CXCR2	5′‐CTCACAAACAGCGTCGTAG‐3′	5′‐AGGTTCTCTGAGTGGCATGG‐3′	5′‐FAM‐TGCCCTCTATTCTGCCAGAT‐BHQ1‐3′
B2MG	5′‐GGTCTTTCTGGTGCTTGTCT‐3′	5′‐AATGTGAGGCGGGTGGAA‐3′	5′‐HEX‐CAAGTATACTCACGCCACCCAC‐BHQ1‐3′

### 2.5 Biochemical assays

The concentration of Malondialdehyde (MDA) in the heart, aorta, and plasma of the experimental groups was determined according to the Mihara and Uchiyama method (Uchiyama & Mihara, 1978). This procedure assesses the final products of polyunsaturated fatty acids peroxidation, such as lipoperoxides like MDA in tissue homogenates (Uchiyama & Mihara, 1978). The MDA level serves as an indicator of tissue lipid peroxidation. In this method, MDA present in 1 mL of aorta or heart tissue homogenate at 100°C reacts with 2 mL of a mixture containing 0.0075 g thiobarbituric acid (TBA), 0.04 mL hydrochloric acid (HCL), and 0.3 g trichloroacetic acid (TCA). After 35–45 min, a reddish complex (absorbance peak: 535 nm) is produced, which serves as an index for MDA concentration, determined by colorimetric methods.

According to Sedlak and Lindsay's method (Sedlak & Lindsay, 1968), thiol groups in certain of the intra‐ or extracellular antioxidants can react with 2,2′‐dinitro‐5,5′‐dithiodibenzoic acid (DTNB), producing a yellowish complex that is detectable and measurable. In this method, the initial absorbance of a mixture of aorta or heart homogenate (0.05 mL) plus 1 mL of Tris‐ethylenediaminetetraacetic acid (EDTA) solution (30 mM Tris, 3 mM EDTA, PH 8.2) was measured at 412 nm, representing the peak absorbance of the yellowish complex. The absorbance reading was repeated after adding 20 μL of 10 mM DTNB to the mixture and allowing it to incubate at room temperature (25°C) for 15 min.

Superoxide dismutase (SOD) is an antioxidant enzyme that catalyzes the dismutation of the superoxide radical into oxygen and hydrogen peroxide (H_2_O_2_). For SOD activity assessment, the method by Madesh and Balasubramanian (1998) was used. Pyrogallol and tetrazolium dye, 3‐ [4, 5‐dimethyl thiazol‐2‐yl] 2, 5‐diphenyltetrazolium bromide (MTT) were added to aorta or heart tissue homogenate. Tissue SOD destroys superoxide radicals produced by pyrogallol oxidation. In this method, the reduction of tetrazolium dye, characterized by a peak absorbance at 535 nm, indicates SOD activity.

Catalase (CAT) is an antioxidant enzyme that catalyzes the decomposition of H_2_O_2_ to water and oxygen. CAT activity was assessed according to the Aebi method (Aebi, 1984), based on the decline in absorbance of the mixture of aorta or heart tissue homogenate and H_2_O_2_ (30 mM) due to H_2_O_2_ decomposition by tissue CAT at 240 nm. The amount of CAT that can degrade 1 μmol of H_2_O_2_ in 1 minute is defined as one unit (U) of the enzyme.

Plasma GSH was assessed according to Ellman's modified method by Hissin and Hilf (1976). In this method SH groups of GSH react with Ellman's reagent, resulting in the formation of a reduced form of the reagent. The reduced form of 2‐nitro‐5‐mercaptobenzoic acid appears yellow and has an absorption peak at 405 nm. GSH concentrations (μmol/L) were calculated using a calibration curve, which was established by measuring absorbance across a concentration range of 0.001–0.1 mM of standard GSH.

The determination of NO levels was performed using the Griess reagent (Promega kit, USA) (Navarro‐Gonzálvez et al., 1998). This method detects nitrates/nitrites (nmol/L or nmol/g tissue) produced from unstable NO. A calibration curve was generated by measuring absorption across a concentration range of 0.5–10 μM of NaNO2 with an absorption peak of 550 nm.

### 2.6 Quantitative real‐time reverse transcription‐polymerase chain reaction

Total cellular RNA extraction from aorta and heart tissues was accomplished following the manufacturer's instructions for the Genet Bio kit. Superscript II reverse transcriptase (RT) and hexamer primers (Table 1) were utilized for cDNA synthesis.

Quantitative RT‐PCR was performed on a Corbet Real‐time PCR detection system (Rotor Gen 6000) using Real Q plus Master Mix for Probe (Bio‐Rad). Amplification reactions were conducted using 20 μL mixtures containing each primer at a concentration of 10 μM (0.5 μL), 1 μL of probe at a concentration of 10 μM, 2 μL of cDNA, and 10 μL of Real Q plus Master Mix 1X. The specific primer and probe sequences are as follows:

The PCR steps included Step 1: initial heat‐denaturing at 95°C for 15 min followed by 40 cycles of 95°C for 30 s, 60°C for 10 s, and 72°C for 10 s. Step 2: The expression of the target gene relative to beta‐2‐microglobulin (B2MG) as an endogenous control gene was calculated using the threshold cycle difference ΔΔCT = ΔCT sample – ΔCT control method (Livak & Schmittgen, 2001), where the relative expression is given by 2^(−ΔΔCT) = 2^(−(ΔCT sample – ΔCT control)). All gene expressions were represented as fold changes relative to the control.

### 2.7 Heart histopathological evaluation

For histopathologic examination, heart apex tissues fixed in formalin were embedded in paraffin, and paraffin casting sectioned (4 μm) were prepared using the Autotechnicon tissue processor. The hematoxylin and eosin (H&E) staining protocol was employed for tissue staining. Pathological changes, including fibrosis, inflammation, and necrosis in the heart tissues of control and infected groups, were assessed. Changes were scored by a qualified pathologist who was blinded to the groups according to the following criteria: (A) no changes = 0, (B) mild changes = 1, (C) severe changes = 2.

### 2.8 Statistical analysis

Data analysis was conducted using SPSS version 16. Qualitative variables were assessed using the Pearson Chi‐Square test. Quantitative data were presented as mean ± SD. The normality of distribution was initially tested using the Kolmogorov–Smirnov method. One‐way ANOVA was applied for parametric data to compare means across different tissues, while the Kruskal–Wallis test was utilized for non‐parametric data. To compare HTLV‐1 infected subjects and controls, the Mann–Whitney *U* test was used for non‐parametric variables, and the Student *t*‐test was applied for parametric variables. A *p*‐value of less than 0.05 was considered statistically significant.

## 3 RESULT

### 3.1 The provirus load (PVL) in PBMCs, splenocytes, and mesenteric nodes

The mean PVL in mesenteric nodes was higher than in PBMCs and splenocytes, although this difference was not statistically significant (Table 2).

**TABLE 2:** Comparison of proviral load (PVL) between PBMCs, splenocytes, and mesenteric nodes in the HTLV‐1‐infected group.

Tissue	PVL
PBMC	33 ± 29.4
Mesenteric nodes	200.77 ± 171.6
Splenocytes	11 ± 5

*Note*: Data are presented as mean ± SD, *n* = 7.

### 3.2 Effect of HTLV‐1 infection on MDA, total thiol, CAT, and SOD in the aorta

**FIGURE 1:**
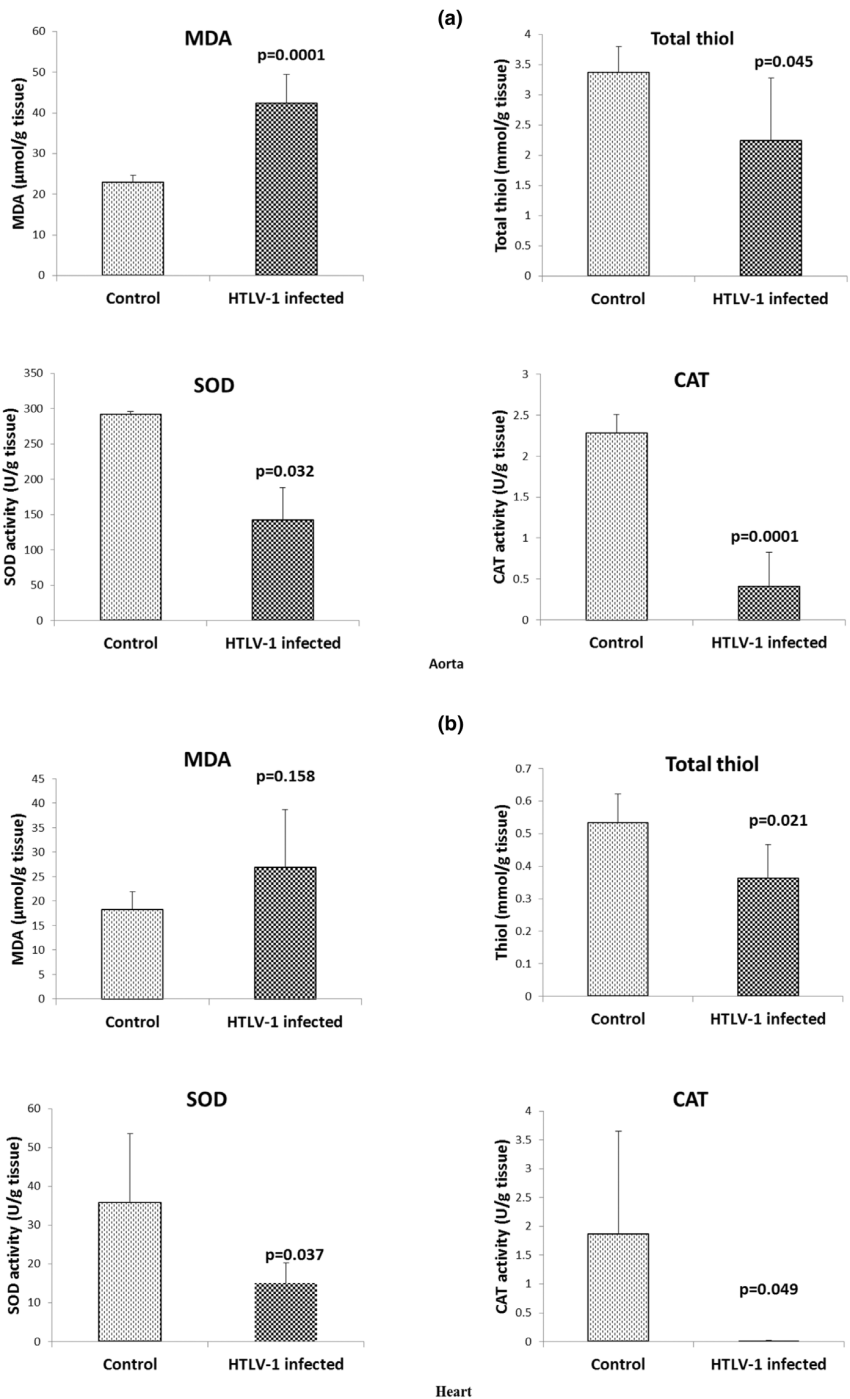
The malondialdehyde (MDA), total thiol levels, catalase (CAT), and superoxide dismutase (SOD) activity of aorta (a) and heart (b) in the control and HTLV‐1‐infected groups. The data are expressed as mean ± SD, *n* = 7. *p* values are compared to the control group, using independent sample *t*‐test.

The MDA level in the aortic tissue of the HTLV‐1‐infected groups was significantly higher than that in the control group (Figure 1a, *p* < 0.001). The levels of total thiol (*p* < 0.05), CAT (*p* < 0.001), and SOD (*p* < 0.05) activity in the aorta of the HTLV‐1‐infected group were lower than those in the control group (Figure 1a).

### 3.3 Effect of HTLV‐1 infection on MDA, total thiol, CAT, and SOD in the heart

An insignificant elevation in the MDA level within the heart tissue of the HTLV‐1‐infected group was observed compared to the control (Figure 1b). The total thiol level, as well as CAT and SOD activity in the heart of the HTLV‐1‐infected group were lower than those in the control group (Figure 1b, *p* < 0.05).

### 3.4 Effect of HTLV‐1 infection on CCR1, CCR2, CXCR2, and eNOS gene expression in the aorta and heart tissues

**FIGURE 2:**
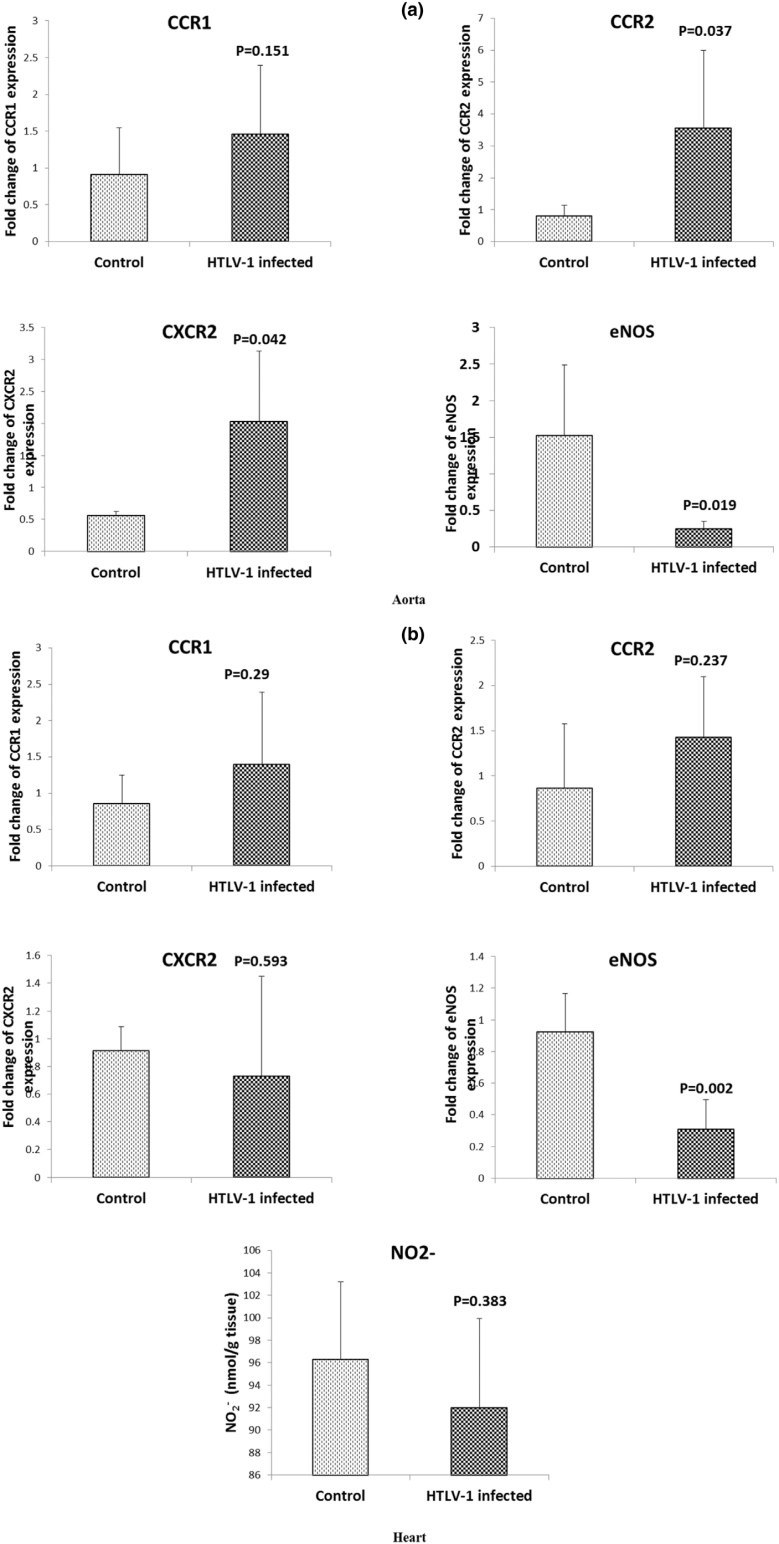
The mRNA expression of CCR1, CCR2, CXCR2, and eNOS of aorta (a) and heart (b) tissues as determined by QRT‐PCR in the control and HTLV‐1‐infected groups. Data were normalized to B2MG mRNA and represent the fold change relative to the control. Figure 2b also represented the nitrite (NO_2_^−^) concentration (nmol/g tissue) of heart tissue in the control and HTLV‐1‐infected groups. Data are expressed as mean ± SD, *n* = 7. *p* values were compared to the control group using independent sample *t*‐test.

An insignificant increase in CCR1 expression was noted in both the aorta (Figure 2a) and heart (Figure 2b) tissues of the HTLV‐1‐infected group. However, CCR2 expression in the aortic tissue of the HTLV‐1‐infected group was significantly higher than that of the control group (*p* < 0.05, Figure 2a). An insignificant elevation in CCR2 expression within the heart tissue of the HTLV‐1‐infected group was also observed (Figure 2b).

QRT‐PCR revealed that HTLV‐1 infection increased the expression of CCR1 and CCR2 1.6 and 4.48‐fold, respectively, in aortic tissue (Figure 2a). Additionally, CCR1 and CCR2 expression in the hearts of infected mice increased by 1.64‐fold in response to HTLV‐1 infection (Figure 2b).

The results also showed that the infection to HTLV‐1 significantly increased CXCR2 expression in the aortic tissue compared to the control group (*p* < 0.05, Figure 2a). A negligible decrease in CXCR2 expression within the heart tissue of the HTLV‐1‐infected group was noted (Figure 2b). The expression of eNOS in the aorta (*p* < 0.05, Figure 2a) and heart (*p* < 0.01, Figure 2b) tissues of the HTLV‐1‐infected group was lower than that of the control group.

### 3.5 Effect of HTLV‐1 infection on NO concentration in the heart tissue

The NO concentration in the heart tissue of the HTLV‐1‐infected group was lower than that in the control group, although this difference was not statistically significant (Figure 2b).

### 3.6 Effect of HTLV‐1 infection on plasma lipids, CK, GSH, total thiol

**FIGURE 3:**
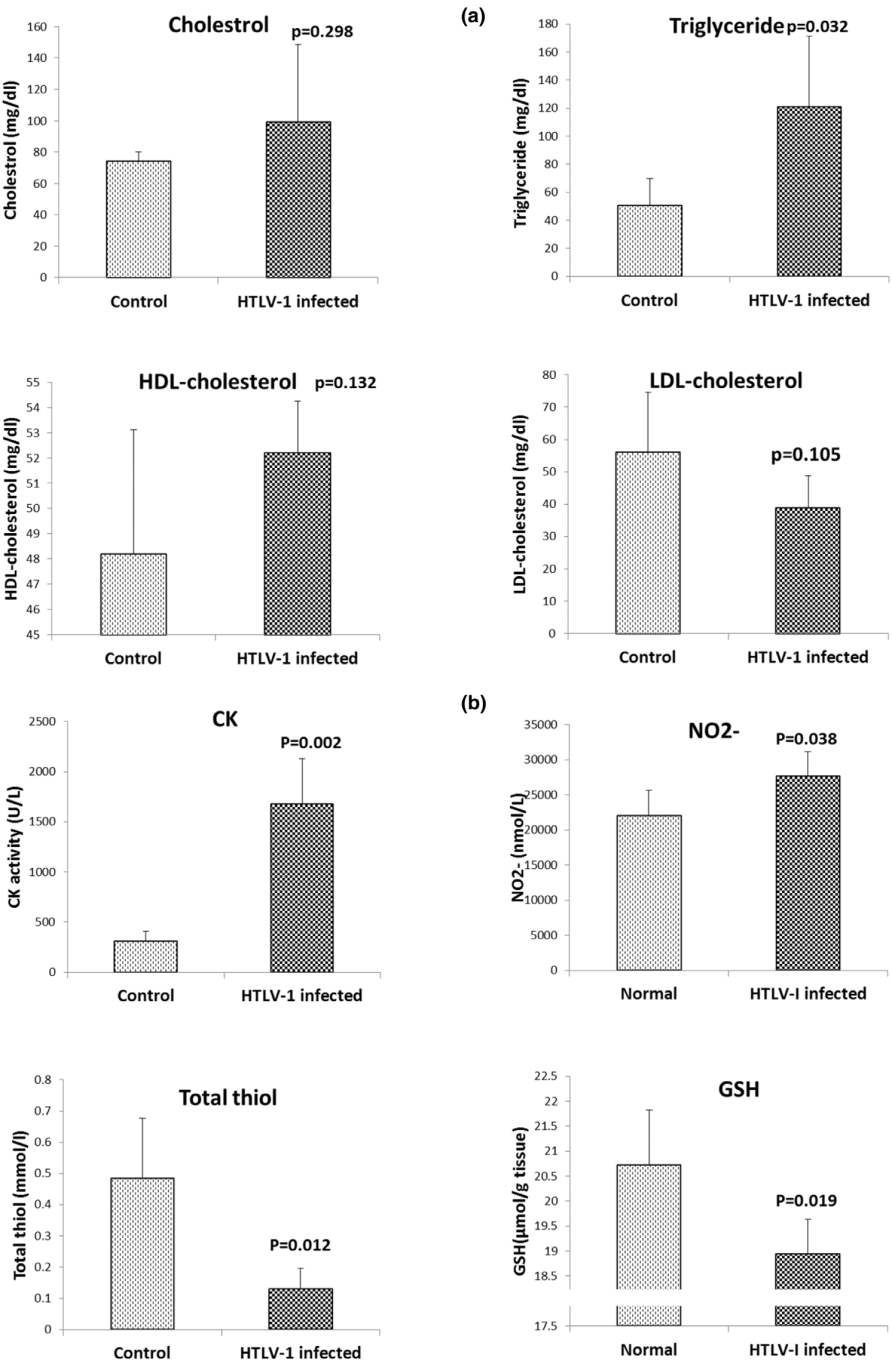
Levels of cholesterol, triglyceride, LDL‐cholesterol, HDL‐cholesterol (a), total thiol, nitrite (NO^−^_2_), glutathione (GSH) and creatinine phosphokinase activity (CK) (b), in plasma of the control and HTLV‐1‐infected groups. The data are expressed as mean ± SD, *n* = 7. *p* values are compared to the control group using independent sample *t*‐test.

Plasma triglyceride levels in the HTLV‐1 infected group were significantly higher than those of the control group (Figure 1b, *p* < 0.05). However, the alterations in plasma cholesterol, LDL‐cholesterol, and HDL‐cholesterol levels were not significant when comparing the HTLV‐1 infected group to the control (Figure 3a).

The CK (*p* < 0.01, Figure 3b) and NO (*p* < 0.05, Figure 3b) levels in the plasma of the HTLV‐1 infected group were higher compared to the control group. Furthermore, GSH and total thiol levels in the plasma of the HTLV‐1 infected group were lower than in the control group (*p* < 0.05, Figure 3b).

### 3.7 Effect of HTLV‐1 infection on heart histopathology

**FIGURE 4:**
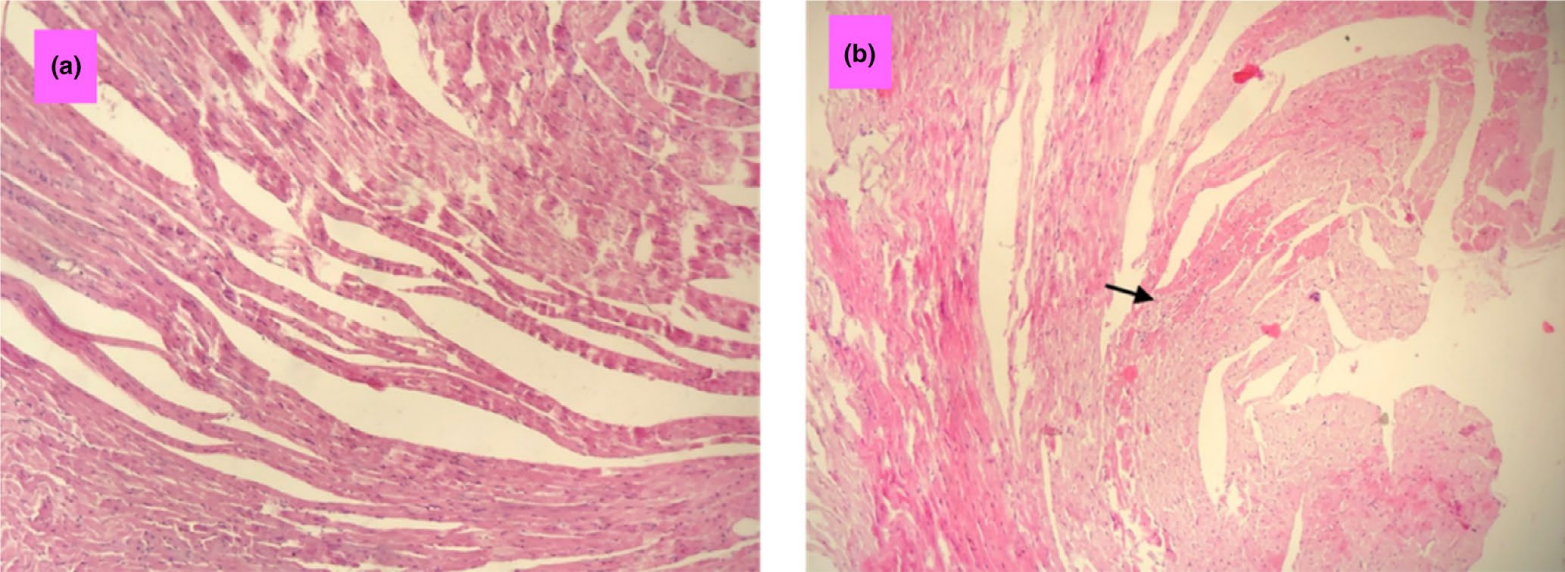
Photographs of apex heart specimen in the control (a) and HTLV‐1‐infected groups (b), (magnification for each group; 40 × 10).

Histological examination of heart tissues from both groups revealed mild inflammation in the heart tissues of the HTLV‐1 infected group compared to the control group (*p* < 0.05, Figure 4, Table 3). No fibrosis or necrosis was observed in the heart tissues of either group.

**TABLE 3:** Pathological finding scores in the control and HTLV‐1 infected groups.

Scores in groups		
Pathological findings	Control	HTLV‐1‐infected
Fibrosis	0.00 ± 0.00	0.00 ± 0.00
Inflammation	0.14 ± 0.18	0.85 ± 0.69 *p* = 0.033
Necrosis	0.00 ± 0.00	0.00 ± 0.00

*Note*: The data is expressed as mean ± SD, *n* = 7. *p* values compared to the control group, using independent sample *t*‐test.

## 4 DISCUSSION

In this study, proviral DNA was detected using the TaqMan Real‐time PCR method in the splenocytes, mesenteric nodes, and PBMCs of BALB/c mice 2 months after MT‐2 injection (IP). Additionally, the PVL in the mesenteric nodes was higher than that in PBMCs and splenocytes, although this difference was not statistically significant. Fang et al. identified HTLV‐1 DNA in the spleen, mesenteric nodes, and thymus of C3H/HeJ mice 15 weeks post‐MT‐2 injection (Fang et al., 1998). Yari et al. demonstrated overexpression of Tax, a regulatory protein of HTLV‐1, in PBMCs of HAM/TSP patients compared to HTLV‐1 carriers using the TaqMan Real‐time PCR method (Yari et al., 2014). Blood and lymphoid tissue PVL in BALB/c mice injected with MT‐2 i.p. were higher than in those injected i.v. (Tanaka et al., 2011). In the Nagai study, the PVL in T lymphocytes of HAM/TSP patients was reported to be approximately 64 copies per 100 cells (Nagai et al., 2001).

In this study, HTLV‐1 infection resulted in a reduction of total thiol levels in aorta, heart, and plasma, a decrease in SOD and CAT activities in aorta and heart, an elevation of MDA levels in the aorta, and a reduction of GSH in plasma. Our results indicated that HTLV‐1 infection attenuates both enzymatic and non‐enzymatic antioxidant defenses and increases lipid peroxidation in the cardiovascular system. Shomali et al. reported that HTLV‐1 infected patients experienced a reduced total antioxidant capacity (Shomali et al., 2015). Moreover, our previous research indicated elevated levels of oxidative stress due to HTLV‐1 infection in the central nervous system (Moghadam et al., 2018). Previous studies have reported that antioxidant agents such as ascorbic acid (vitamin C) and green tea are beneficial in HTLV‐1 infection (Kataoka et al., 1993; Moens et al., 2012; Sonoda et al., 2004). Kinjo et al. demonstrated that Tax expression in primary human cells induces reactive oxygen species (ROS) production (Kinjo et al., 2010). Similarly, Takahashi et al. reported that HTLV‐1 Tax stimulates ROS production in T‐lymphocytes (Takahashi et al., 2013).

The expression level of the eNOS gene in the heart and aorta of the HTLV‐1 infected group significantly decreased compared to the control group. Additionally, NO plasma concentration increased significantly in the HTLV‐1 infected group compared to the control group Additionally, NO in plasma concentration increased significantly in the HTLV‐1 infected group compared to the control group. The increased plasma concentration of NO in HTLV‐1‐infected mice may be due to the expression and activation of iNOS in other tissues such as the kidneys. Baydoun et al. reported that NO production could occur by iNOS in HTLV‐1 infected cells or Tax expressing cells (Baydoun & Ratner, 2014; Salmaninejad et al., 2019).

The results of this study also showed that HTLV‐1 induces the expression of CCR2 and CXCR2 in the aorta. The increased expression of CCR1 and CCR2 on epithelial and endothelial cells is associated with local inflammation (Zhang et al., 2011). Weber reported endothelial cells express CCR2, which may have important implications for inflammatory reactions (Weber et al., 1999). Dzenko's study demonstrated that endothelial CCR2‐mediated transendothelial migration (Dzenko et al., 2005). Additionally, the expression of CCR2 on vascular smooth muscle cells may influence their proliferation and migration capacity (Hayes et al., 1998). Several studies reported that HTLV‐1 infection increases CCR2 and CXCR2 expression on the surface of T lymphocytes via the Tax protein (Higuchi & Fujii, 2009). CXC and CC chemokine receptors on coronary endothelial cells could play a role in endothelial migration during HIV‐1 tissue invasion and angiogenesis (Berger et al., 1999). More recently, Mohammadi et al. clarified that the upregulation of CCR2 in PBMCs of HTLV‐1 infected patients with coronary artery disease exists. They also reported a direct or indirect correlation between HTLV‐1 infection and alteration of lipid profile, chemokine receptors, including CCR1 and CCR2, as well as cytokine secretion in HTLV‐1 infected individuals (Hemmatzadeh et al., 2019; Mohammadi et al., 2019). Tax induced production of cytokines like IL‐2 acts as an atherosclerosis risk factor in HTLV‐1 carriers (Higuchi & Fujii, 2009). Various studies have shown that white blood cells with high CXCR1, CCR2, and CXCR1 exhibit high migration capacitance (Williams et al., 2015).

The study observed a higher proviral load in mesenteric lymph nodes compared to PBMCs and splenocytes, although this difference was not statistically significant. Biochemical assessments of plasma revealed elevated triglyceride and CK levels in HTLV‐1‐infected mice compared to controls. Carvalho et al. reported that HTLV‐1 infection in women is linked to elevated serum triglyceride and very‐low‐density lipoprotein (VLDL‐cholesterol) cholesterol levels, suggesting that viral infections may enhance lipid profiles through receptor interactions as a defense mechanism (Carvalho et al., 2015). In a study using pX transgenic mice, Koizumi et al. demonstrated that patients with rheumatoid arthritis and HTLV‐1 infection have a greater susceptibility to atherosclerosis and cardiovascular diseases, attributed to hyperlipidemia (Babaie et al., 2018; Koizumi et al., 2005). The observed increase in plasma CK levels in infected mice may indicate the onset of inflammatory responses and could be associated with neuromuscular disorders or damage to the cardiovascular system.

The existence of a strong link between oxidative stress and cardiovascular disorders has been acknowledged by several researchers (Arrigo, 1999; Ghiselli et al., 2001). Furthermore, it is expected that reduced endothelial capability in NO production could induce endothelial dysfunction and promote a vascular phenotype more prone to atherogenesis (Förstermann et al., 2017). The critical roles of CCR2 and CXCR2 in further disruptions, such as endothelial dysfunction, smooth muscle cell proliferation, angiogenesis, and WBC infiltration/activation/adhesion highlight the importance of these receptors in vascular atherosclerosis (Speyer & Ward, 2011; Zernecke & Weber, 2009). Therefore, HTLV‐1, as an inducer of oxidative stress, chemokine receptors, and eNOS downregulation in cardiovascular system tissues, may chronically initiate or exacerbate the progression of atherosclerosis and ischemic heart disease in infected individuals. However, more research, including studies on animals with prolonged infections examining the alteration of adhesion molecules and cytokines, is needed to understand the connection between HTLV‐1 infection and cardiovascular injuries.

## 5 CONCLUSION

This study clearly highlights the significant impact of HTLV‐1 infection on inflammatory and oxidative stress markers in cardiovascular tissues. The findings indicate that HTLV‐1 infection leads to a substantial increase in levels of triglycerides and creatine phosphokinase. The elevation in triglyceride levels may suggest metabolic disturbances that arise following HTLV‐1 infection. Additionally, the high levels of creatine phosphokinase can serve as an indicator of cardiac tissue damage in patients infected with this virus. Furthermore, these findings underscore the importance of further investigating the relationship between HTLV‐1 infection and cardiovascular diseases. Understanding this connection could aid in the development of effective preventive and therapeutic strategies for patients with HTLV‐1 infection. Ultimately, this study emphasizes the necessity for more research into the long‐term effects of HTLV‐1 infection on heart and vascular health, enabling a better understanding of the underlying mechanisms of these diseases and the formulation of appropriate management strategies.

## FUNDING INFORMATION

This study was financially supported by the Research Council of Mashhad University of Medical Sciences, Mashhad, Iran.

## CONFLICT OF INTEREST STATEMENT

The authors declare that they have no conflict of interest.

## ETHICS STATEMENT

All experimental procedures were conducted in accordance with the guiding principles of animal care and use, as approved by the Mashhad University of Medical Sciences Ethical Commission (IR.MUMS.fm.REC.1394.171). This study was financially supported by the Research Council of Mashhad University of Medical Sciences, Mashhad, Iran (940111).

## Data Availability

Data available within the article.
